# The effect of GABAmimetics on the duration of immobility in the forced swim test in albino mice

**DOI:** 10.3402/ljm.v9.23480

**Published:** 2014-02-18

**Authors:** Najwa Ahmed El zahaf, Abdalla Salem Elhwuegi

**Affiliations:** Faculty of Pharmacy, Department of Pharmacology and Clinical Pharmacy, Tripoli University Tripoli, Libya

**Keywords:** GABAmimetics, forced swim test, diazepam, vigabatrin, zolpidem, alprazolam

## Abstract

**Objectives:**

Studies regarding the role of gamma aminobutyric acid (GABA) in depression are conflicting. Therefore, it was decided to examine the effect of different drugs that enhance the GABA system on the time of immobility induced by the forced swim test (FST).

**Materials and methods:**

Adult albino mice were divided into several groups of six animals. Each group received an intraperitoneal injection of either imipramine (10, 20, or 30 mg/kg), diazepam (0.5, 1, or 2 mg/kg), vigabatrin (100, 200, or 300 mg/kg), zolpidem (2.5, 5, or 10 mg/kg), or alprazolam (1, 2.5, or 5 mg/kg). Control groups received the appropriate vehicle. One hour after injection, the duration of immobility was measured for 5 min in the FST. The percentage change in the duration of immobility from the control was calculated for each group. The statistical test of the difference between the treated and the control groups was calculated using unpaired Student's *t*-test.

**Results:**

Imipramine produced a significant dose-dependent decrease in the duration of immobility (78, 74, and 56%, respectively). Different doses of diazepam, vigabatrin, and zolpidem produced a significant increase in the duration of immobility (119, 126, and 128%), (116, 124, and 128%), and (108, 109, and 119%), respectively. The two low doses of alprazolam produced a significant increase (115 and 120%), while the high dose produced a significant decrease in the duration of immobility (74%).

**Conclusion:**

Increasing central GABAergic activity by different mechanisms has resulted in a depressant-like activity measured as an increase in the duration of immobility in the FST model of depression.

Depression is a common mental disorder affecting millions of people worldwide and is also a leading cause of disability. At its worst, depression can lead to suicide, a fact associated with the loss of about 850,000 lives every year (1).

Many neurotransmitters have been suggested to be involved in the mechanisms of depression and antidepressant drugs. A strong role for the monoamines, serotonin (5-hydroxytryptamine, 5-HT) and noradrenaline (NA) in major depression has been proposed (2). On the other hand, the role of gamma aminobutyric acid (GABA) is still not well recognized and several studies are showing conflicting findings regarding any crucial role for GABA in the pathophysiology of depression. It was reported that small doses of GABA or its agonists (muscimol, baclofen, sodium valproate, piracetam, and fengabine) decreased a forced swimming-induced immobility period, while higher doses enhanced the immobility period (3). Conversely, diazepam, flurazepam, pentobarbital, and phenobarbital enhanced the immobile behavior in a dose-dependent manner (4). Clinically, it has been suggested that reduced concentrations of GABA and altered expression of GABA_A_ receptors are common abnormalities observed in major depressive disorders (5). However, currently available GABA potentiating drugs are ineffective as antidepressants (5). On the basis of these findings, it was decided to examine if enhancing central GABA by different mechanisms has a direct role in an established model of animal depression, the forced swim test (FST). Vigabatrin acts as an irreversible inhibitor for the enzyme GABA-Transaminase (GABA-T) responsible for GABA metabolism, which would lead to an increase in brain GABA levels (6). Diazepam (7) and alprazolam (8) are positive allosteric modulators of α1-, α2-, α3-, and α5-containing GABA_A_ receptor subunits, potentiating GABA-mediated chloride conductance, enhancing GABA-mediated inhibitory synaptic events. Zolpidem is a selective agonist for the α1-subunit of GABA_A_ receptors (9). Imipramine, which acts by inhibiting the neuronal re-uptake of NA and serotonin, is included as a standard antidepressant drug (2). The effect of different doses of these drugs on the duration of immobility in the FST using albino mice is reported in this article.

## Materials and Methods

### Animals

Adult male albino mice weighing 25–40 g at the time of testing were used. The animals were obtained from the animal house of the Faculty of Pharmacy, Tripoli University, 1 week before the experiment. They were housed in cages of six each and allowed free access to standard rodent food pellets and water. The animals were kept at constant room temperature (20–25°C), and 12 h dark/light cycle. All experiments were conducted between 10 am and 12:00 pm. Each experimental group consisted of six animals per dose and each animal was used only once. The ethical committee of the Faculty of Pharmacy, Tripoli University, approved the experimental protocol.

### Drugs

Imipramine ampoules (from Polfa, Poland) were diluted with normal saline. Vigabatrin oral powder (Aventis, France) was prepared in normal saline. Diazepam (Roch, Switzerland), alprazolam (Sigma-Aldrich), and zolpidem (Sigma-Aldrich) were suspended in 1% Tween 80 in saline solution.

### Treatments

The animals were divided into several groups of six animals each. Each mouse received an intraperitoneal injection of either imipramine (10, 20, or 30 mg/kg), diazepam (0.5, 1, or 2 mg/kg), vigabatrin (100, 200, or 300 mg/kg), zolpidem (2.5, 5, or 10 mg/kg), or alprazolam (1, 2.5 or 5 mg/kg) in a volume of 5 ml/kg 60 min prior to the FST. Control group was given the corresponding vehicle.

### Forced swim test

The FST procedure was essentially similar to that described by Porsolt et al. (10). The test was modified by using a Plexiglas cylinder of larger diameter (height 40 cm; diameter 20 cm) as this was reported to increase the predictive validity of the mouse FST (11). The depth of the water in the cylinder was kept at 15 cm above the bottom of the cylinder so that the animal was forced to either swim or float without its hind limbs or tail touching the bottom.

Each mouse was placed inside the cylinder containing water maintained at 22–25°C and the duration of immobility was measured for 5 min. The water was replaced between each test. The mouse was judged to be immobile whenever it remained floating passively in water making only those movements necessary to keep its head just above the water surface (11). The duration of immobility was measured manually using a stopwatch.

### Data presentation and statistical analysis

The mean duration of immobility was calculated for each group in seconds±standard error. The percentage changes from the correspondent control (vehicle treated) group were also calculated. Data generated from the above studies were statistically analyzed with Excel software. Unpaired Student's *t*-test (two samples assuming equal variances) was used to determine the difference between the treated group and the control group that received the vehicle. A *P* value of <0.05 was considered statistically significant.

## Results

**Fig. 1:**
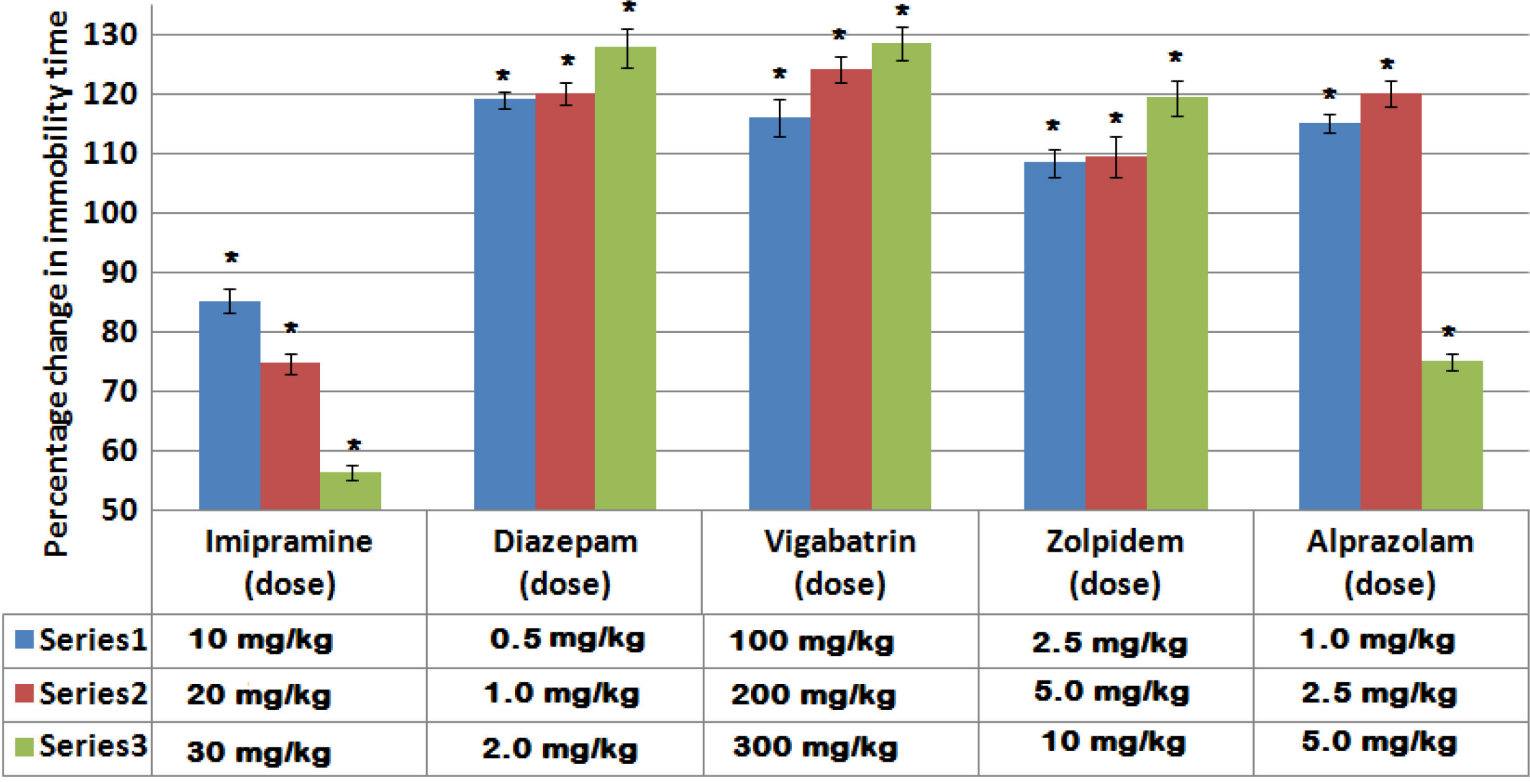
Effects of drug treatments on the percentage change in the immobility time in FST. Columns represent Mean±S.E. Drugs were administered 1 h before the test session and the duration of immobility was measured for 5 min. *P<0.001 as compared to control group using unpaired Student's *t*-test (two samples assuming equal variances) (*n*=6).

Figure 1 shows the effect of the selected drugs on the duration of immobility in FST. Imipramine produced a significant dose-dependent reduction in the duration of immobility with a maximum reduction of 56.3% from the control group. Ascending doses of diazepam, vigabatrin, and zolpidem produced a significant increase in the duration of immobility with a maximum increase of 128, 128.5, and 119.3% from the corresponding control groups, respectively. On the contrary, alprazolam produced a dual effect on the duration of immobility, where the low doses produced a statistically significant increase in the duration of immobility with a maximum of 120% from the control group, while the highest dose produced a statistically significant reduction in the duration of immobility (74.9% from the control group).

## Discussion

The current study uses a well-established model of animal depression in order to examine if the selected drugs that act through the GABA system have any antidepressant-like activity.

Consistent with many previous findings, imipramine produced a significant dose-dependent antidepressant-like effect in the mouse FST. The antidepressant action of imipramine is thought to be due to reuptake inhibition of the monoamines serotonin and NA (2). Imipramine has also been suggested to produce its antidepressant action through the interference with the GABAergic system, where the antidepressant effect of imipramine was enhanced by picrotoxin (GABA_A_ receptor antagonist), suggesting a possible role for GABA in the antidepressant effect of imipramine (12).

Except for the high dose of alprazolam, it was found that all of the selected drugs that enhance central GABA activity produced a significant increase in the duration of immobility in FST (i.e. depressant-like action). These results are in a good agreement with those reported by Nagatani et al. (4), but contradictory to the proposal suggested by Luscher et al. (5). The obtained results can be explained on the basis of the complexity of GABA_A_ receptors and its relationship to the monoamines.

GABA_A_ receptors are formed as pentameric combinations of homologous subunits (α1–6, β1–3, γ1–3, δ, ɛ, θ, π, ρ1–3), with receptors formed by α, β and γ subunits being the most common. The α subunits provide the largest diversity where GABA_A_ receptors are most frequently classified by their α subunits (13). Each of these subunits was suggested to be related to certain function and therapeutic applications. It was reported that modulators of α2/α3 GABA_A_ receptors subunits, such as TPA023, had shown clinical proof as novel anxiolytics and antidepressants (14). The α5-subunit of the GABA_A_ receptors has been suggested to represent the ‘cognition’ subtype based upon the preferential localization of these receptors within the hippocampus and the well-established role of the hippocampus in learning and memory (15). The function of α2-containing GABA_A_ receptors was also suggested to be involved in antidepressant-like properties (16). From these reported actions of the GABA subunits, it is therefore logical to speculate that the depressant-like activity of the drugs used in the present study might have resulted from the stimulation of the α1 subunit of the GABA_A_ receptors either selectively (like zolpedim) or non-selectively (like diazepam). This would explain the depressant-like action of all the GABAmimetics used in this study.

The dual action induced by alprazolam is more intricate to explain. However, at the small doses, alprazolam might have acted as a full agonist at all subunits of the GABA_A_ receptors especially the α1-subunit enhancing GABA mediated inhibitory effect, thus prolonging the duration of immobility in the FST. Conversely, the high dose of alprazolam might have acted as an agonist on the α_2_ adrenergic auto-receptors resulting in an enhancement of 5-HT release, producing an antidepressant effect in the FST. This suggestion is supported by the findings that alprazolam's hippocampal 5-HT/NA interaction is similar to clonidine's 5-HT/NA action at α_2_ adrenergic auto-receptors, resulting in enhanced 5-HT release in hippocampus, an action which was not produced by diazepam (17).

Another point of view is the relationship between GABA and the monoamine system. There is a general agreement that central monoamines are involved in the etiology of depression (2). Many previous animal studies reported a complex interaction between GABAergic and monoaminergic transmissions, especially within the raphe nuclei which receives in particular GABAergic input (18). As expected, the GABAergic input onto 5-HT neurons reduced the firing of these neurons (18). Moreover, the application of GABA agonists, such as muscimol, in dorsal and medial raphe nuclei inhibited 5-HT neuronal release (19). Accordingly, it is possible to speculate that high basal GABA activity might have led to a reduced level of serotonergic transmissions in certain brain areas, which would result in a depressive-like activity, measured as an increase in the time of immobility in the experiments reported in this research work.

Therefore, it can be concluded that increasing central GABAergic activity by different mechanisms will result in a depressant-like activity most likely through the α1 subunit of GABA_A_ receptors.

## Limitation of this study

This study would have provided more information if selective an antagonist at the α1 subunit of the GABA_A_ receptors was included.
